# Diagnostic accuracy of the Vesical Imaging Reporting and Data System for muscle-invasive bladder cancer and its role in reducing repeat transurethral resection of bladder tumor: A systematic review

**DOI:** 10.14440/bladder.0022

**Published:** 2025-11-12

**Authors:** Debanjan Nandi, Surya Kant Kumar, Arshad Hasan, Vinod Kumar

**Affiliations:** 1 Department of Radiology, All India Institute of Medical Sciences, Kalyani 741245, West Bengal, India; 2 Department of Urology, Narayan Medical College and Hospital, Jamuhar 821305, Rohtas, India; 3 Department of Urology, Patna Medical College and Hospital, Patna 800004, Bihar, India; 4 Department of Urology, All India Institute of Medical Sciences, Kalyani 741245, West Bengal, India

**Keywords:** VI-RADS, Bladder cancer, Muscle-invasive bladder cancer, Multiparametric magnetic resonance imaging, Transurethral resection of bladder tumor, Diagnostic accuracy, Specificity

## Abstract

**Background:**

The Vesical Imaging Reporting and Data System (VI-RADS) has been introduced as a standardized tool for assessing multiparametric magnetic resonance imaging (MRI) in the pre-operative staging of bladder cancer.

**Objective:**

The present systematic review evaluates the efficacy of VI-RADS in diagnosing muscle-invasive bladder cancer (MIBC) compared with established diagnostic techniques. A systematic review was conducted according to PRISMA guidelines using PubMed, Scopus, and Web of Science. Studies assessing VI-RADS for MIBC diagnosis were included if they reported sensitivity, specificity, area under the curve, and comparisons with conventional approaches. Study characteristics, sample size, VI-RADS cutoff, diagnostic performance, and risk of bias were evaluated. Thirteen studies (sample sizes ranging from 18 to 340) demonstrated high diagnostic performance, with sensitivity ranging from 78% to 100%, specificity from 73% to 96%, and AUC from 0.86 to 0.96. VI-RADS outperformed standard assessment in reducing transurethral resection of bladder tumor (TURBT)-related understaging. A forest plot showed variability in sensitivity, influenced by radiologist experience, magnetic resonance imaging protocols, and study design. The risk of bias was moderate-to-low.

**Conclusion:**

VI-RADS is an effective non-invasive tool for detecting MIBC and plays an important role in pre-operative decision-making. It may reduce the need for repeat TURBT. Standardization of MRI protocols and radiologist training is vital to improve diagnostic reliability.

## 1. Introduction

Bladder cancer is one of the most common malignancies worldwide, with approximately 549,000 new cases and 200,000 deaths annually.^1^-^2^ The predominant histological type is urothelial carcinoma, which is classified as non-muscle-invasive bladder cancer (NMIBC) and muscle-invasive bladder cancer (MIBC).^3^ NMIBC is confined to the mucosal layer and generally has a favorable prognosis, whereas MIBC, which involves invasion into the detrusor muscle, requires radical cystectomy.^4^ Accurate staging is indispensable because treatment decisions, particularly for MIBC, determine the need for neoadjuvant chemotherapy and bladder-sparing therapies.

Transurethral resection of bladder tumor (TURBT) with subsequent histopathological analysis is the gold standard for staging bladder cancer.^5^ Nevertheless, TURBT may not always accurately determine muscle invasion, potentially leading to either understaging or overstaging, particularly in cases of large or multifocal tumors or incomplete resection. Cystoscopy is the main diagnostic modality; however, it has limitations in detecting muscle invasion, especially in anatomically challenging.^6^

The Vesical Imaging Reporting and Data System (VI-RADS) has recently been proposed as a standardized reporting system for multiparametric magnetic resonance imaging (mpMRI) in bladder cancer. VI-RADS employs a five-point scoring system based on T2-weighted imaging (T2WI), diffusion-weighted imaging (DWI), and dynamic contrast-enhanced imaging (DCEI) to evaluate the likelihood of muscle invasion.^7^ This non-invasive imaging approach improves diagnostic precision and provides in-depth tumor localization without exposure to ionizing radiation. This systematic review was conducted to assess the diagnostic value of VI-RADS in detecting MIBC and to evaluate its clinical impact on pre-operative decision-making and staging accuracy compared to conventional imaging approaches.

## 2. Methods

### 2.1. Eligibility criteria

This systematic review included studies assessing VI-RADS for diagnosing MIBC and reporting diagnostic metrics such as sensitivity, specificity, positive predictive value (PPV), negative predictive value (NPV), accuracy, and area under the curve (AUC). Studies were eligible if they compared VI-RADS with conventional diagnostic methods. Exclusion criteria included studies without diagnostic comparisons, non-human studies, non-English publications, studies focused exclusively on NMIBC, and studies not addressing MIBC detection.

### 2.2. Search strategy and information sources

A comprehensive literature search was conducted across multiple electronic databases, including PubMed, Scopus, and Web of Science, to identify studies evaluating the diagnostic performance of VI-RADS in detecting MIBC. The search covered publications from January 2018 to December 2023. The following search terms and Boolean operators were used: (“VI-RADS” OR “vesical imaging reporting and data system”) AND (“bladder cancer” OR “urothelial carcinoma”) AND (“muscle-invasive” OR “MIBC”) AND (“MRI” OR “multiparametric MRI”). Filters were applied to include only original research articles in English involving human subjects. Additional articles were identified through manual reference screening of relevant studies and reviews. The search strategy was tailored to each database, and all retrieved studies were imported into reference management software to eliminate duplicates.

### 2.3. Study selection process

**Figure 1:**
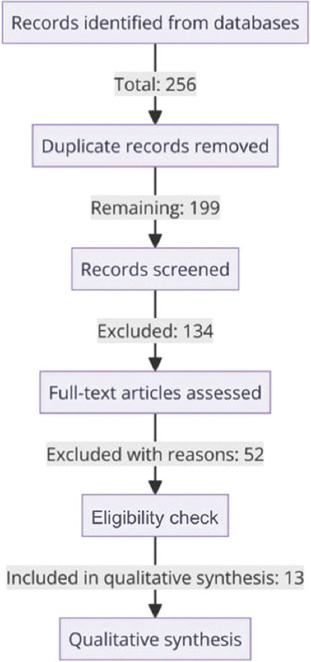
PRISMA flowchart illustrating the systematic review process

The study selection process followed a systematic approach to ensure the inclusion of relevant studies. Initially, the titles and abstracts of the retrieved studies were screened for relevance, and studies that did not meet the inclusion criteria were excluded at this stage. After the initial screening, full-text versions of the remaining studies were reviewed to verify their eligibility based on the inclusion and exclusion criteria. The PRISMA flow diagram was used to document and illustrate the study selection process, including the number of studies retrieved, screened, assessed for eligibility, and ultimately included in the review (Figure 1).

### 2.4. Data collection process

Data were independently extracted from each study by two reviewers to minimize bias and improve accuracy. Any disagreements were resolved through discussion or consultation with a third reviewer. No automation tools were used in the data extraction process.

The key outcomes extracted were diagnostic performance measures (e.g., sensitivity, specificity, PPV, NPV, and AUC) of VI-RADS in detecting MIBC. Results relevant to these outcomes were extracted from all included studies.

Additional variables extracted included study details (e.g., author[s], year of publication, and country of origin), sample size, diagnostic methods (including VI-RADS scoring system and cutoff values), magnetic resonance imaging (MRI) sequences used (e.g., T2WI, DWI, and DCEI), and comparison methods (e.g., TURBT or biopsy). Missing or unclear information was addressed by contacting study investigators when necessary.

### 2.5. Risk of bias assessment

Several potential sources of bias were identified in the included studies. Radiologists with greater experience using VI-RADS tended to achieve higher diagnostic performance, whereas those with less experience showed lower interobserver agreement. However, the moderate to substantial agreement reported across studies suggests that VI-RADS can be effectively used by radiologists with varying levels of expertise. Study design also affects bias; multicenter cohort studies were generally more reliable, whereas single-center studies were more susceptible to selection bias and limited generalizability due to homogeneous populations. Vague descriptions of MRI protocols or differences in MRI scan sequences between centers may have contributed to variability. Sample selection bias was noted in studies involving high-risk populations, such as patients with recurrent NMIBC or those undergoing repeat TURBT, which may reduce the generalizability of the findings. Finally, although VI-RADS is validated, large-scale randomized controlled trials are required to confirm its performance across diverse settings, as some included studies were retrospective or had a small sample size.

### 2.6. Quality assessment

The methodological quality and risk of bias of the included studies were assessed using the Quality Assessment of Diagnostic Accuracy Studies (QUADAS)-2 tool. This tool evaluates potential bias across four key domains: Patient selection, index test, reference standard, and flow and timing. For each domain, studies were classified as having a low, high, or unclear risk of bias. The QUADAS-2 tool enabled assessment of whether the VI-RADS was applied consistently, whether the gold standard diagnostic method was appropriately used, and whether potential biases in patient selection or follow-up were accounted for. Studies identified as having a high risk of bias or significant methodological limitations were given less weight in the final synthesis.

## 3. Results

This review included 13 studies with sample sizes ranging from 18 to 340 patients, with designs varying from small-scale prospective studies to multicenter cohort studies. All studies evaluated the diagnostic performance of VI-RADS using mpMRI to detect MIBC, comparing it with conventional methods such as cystoscopy, TURBT, and biopsy. VI-RADS cutoffs of ≥3 and ≥4 were used. Sensitivity, specificity, and AUC values were extracted, and the study characteristics are summarized in Table 1.

**Table 1:** Summary of study characteristics and diagnostic performance of VI-RADS in detecting muscle-invasive bladder cancer

Study	Sample size	MIBC/non-MIBC	Confirmation method	Diagnostic methods	Sensitivity/specificity/AUC
Kufukihara *et al*.^8^	61	20/41	TURBT	mpMRI, VI-RADS	Sens: 93.8%, Spec: 88.9%
Sakamoto *et al*.^9^	176	54/122	TURBT+cystectomy subset	mpMRI, VI-RADS+ADC values	AUC: 0.94
Ueno *et al*.^10^	96	35/61	TURBT	mpMRI, VI-RADS	Sens: 80–85%, Spec: 73–89%
Del Giudice *et al*.^11^	231	75/156	TURBT	mpMRI, VI-RADS	AUC: 0.94
Jai-ua *et al*.^12^	58	26/32	TURBT	mpMRI, VI-RADS	Sens: 92.3%, Spec: 86.7%
Reddy *et al*.^13^	91	39/52	TURBT	mpMRI, VI-RADS	Sens: 100%, Acc: 91.3%
Islam *et al*.^14^	200	78/122	TURBT+biopsy	mpMRI, VI-RADS	Sens: 97.56%, NPV: 93.33%
Kural *et al*.^15^	91	29/62	TURBT	mpMRI, VI-RADS	Sens: 79.4%, Spec: 94.2%
Wang *et al*.^16^	340	122/218	TURBT+cystectomy subset	mpMRI, VI-RADS	AUC: 0.94
Etxano *et al*.^17^	18	6/12	TURBT	mpMRI, VI-RADS	Sens: 91.7%, Spec: 87.5%
Ghanshyam *et al*.^18^	74	23/51	TURBT	mpMRI, VI-RADS	AUC: 0.90

Abbreviations: Acc: Accuracy; ADC: Apparent diffusion coefficient; AUC: Area under the curve; DCEI: Dynamic contrast-enhanced imaging; DWI: Diffusion-weighted imaging; MIBC: Muscle-invasive bladder cancer; mpMRI: Multiparametric magnetic resonance imaging; NMIBC: Non-muscle-invasive bladder cancer; NPV: Negative predictive value; PPV: Positive predictive value; Sens: Sensitivity; Spec: Specificity; TURBT: Transurethral resection of bladder tumor; T2WI: T2-weighted imaging; VI-RADS: Vesical Imaging Reporting and Data System.

### 3.1. Diagnostic performance of VI-RADS

Overall, the diagnostic performance of VI-RADS was high across all included studies. At a cutoff of ≥3, sensitivity ranged from 78% to 100%, and specificity ranged from 73% to 96%. At a cutoff of ≥4, sensitivity remained high while specificity increased to 85–94%. The PPV for VI-RADS ≥4 was remarkably high, suggesting reliable identification of muscle invasion, and the NPV was also robust, enabling MIBC to be ruled out in low-score cases. AUC values ranged from 0.86 to 0.94 for VI-RADS ≥3 and 0.90–0.96 for VI-RADS ≥4, representing strong diagnostic accuracy. VI-RADS performed better than conventional diagnostic techniques, including in challenging anatomical regions, such as the bladder neck, due to the integration of multiple MRI sequences (T2WI, DWI, and DCEI).

### 3.2. Risk of bias assessment

Several potential sources of bias were identified in the included studies. Diagnostic performance of VI-RADS was influenced by radiologist experience, with less experienced radiologists demonstrating lower interobserver agreement. In terms of study design, multicenter cohort studies were generally more reliable than smaller, single-center studies, which were more prone to selection bias. Variability in MRI protocols and insufficiently described imaging parameters also contributed to heterogeneity. Sample selection bias was noted in studies involving high-risk populations, limiting generalizability. While VI-RADS is well-validated for staging MIBC, its performance in broader populations requires confirmation through large-scale, well-conducted randomized controlled trials. Furthermore, some studies had limited internal validity due to retrospective design and small sample size, which may have introduced bias in the estimation of diagnostic accuracy. Nonetheless, moderate to substantial interobserver agreement was reported in most studies.

**Figure 2:**
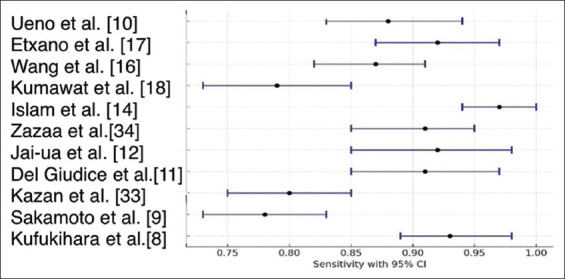
Forest plot of sensitivity across studies for VI-RADS in detecting muscle-invasive bladder cancer Abbreviation: VI-RADS: Vesical Imaging Reporting and Data System.

The forest plot in Figure 2 visually represents the sensitivity of VI-RADS in detecting MIBC across multiple studies, along with their 95% confidence intervals. Sensitivity reflects the ability of VI-RADS to correctly identify patients with MIBC; therefore, higher values indicate a lower false-negative rate. The plot shows variability in sensitivity estimates across the included studies. Studies such as Islam *et al.*,^14^ Ueno *et al.*,^10^ and Del Giudice *et al*.^11^ reported sensitivity values above 0.90 with relatively narrow confidence intervals, indicating strong diagnostic performance with minimal variability. In contrast, studies such as Sakamoto *et al*.^9^ and Jai-ua *et al*.^12^ demonstrated sensitivity values around 0.80 with wider confidence intervals, suggesting greater uncertainty in their estimates.

## 4. Discussion

### 4.1. Key findings

This systematic review demonstrates that VI-RADS is a highly accurate tool for detecting MIBC. Across the included studies, VI-RADS sensitivity ranged from 78% to 100%, while specificity varied between 73% and 96%, confirming its strong diagnostic performance.^19^-^22^ The AUC values, which measure overall diagnostic accuracy, were consistently high (0.86–0.96), indicating reliable differentiation between MIBC and NMIBC.^23^

Compared to conventional diagnostic methods such as cystoscopy and TURBT, VI-RADS demonstrated superior accuracy in assessing muscle invasion. While cystoscopy remains the standard for visual tumor assessment, it lacks the ability to determine the depth of invasion, leading to potential understaging.^24^-^26^ Similarly, although TURBT is the gold standard for obtaining tissue specimens, it carries a risk of understaging of up to 25%, particularly when muscle tissue is absent from the biopsy sample.^27^ In contrast, VI-RADS provides a non-invasive alternative by integrating mpMRI sequences (T2WI, DWI, and DCEI), allowing for precise assessment of muscle invasion before surgery.^28^-^30^

Despite its high diagnostic accuracy, interobserver variability remains a limitation. Several studies reported moderate to substantial interobserver agreement, with kappa values ranging from 0.45 to 0.75, depending on radiologist experience. This suggests that VI-RADS interpretation may be affected by radiologist expertise, emphasizing the need for standardized training to improve consistency.

### 4.2. Clinical implications

The findings from this review suggest that VI-RADS has significant clinical potential for improving bladder cancer staging. One of the key advantages of VI-RADS is its ability to reduce the need for unnecessary repeat TURBT procedures, which are often performed due to concerns about understaging.^2^,^14^ Multiple studies have shown that a VI-RADS cutoff score of ≥3 or ≥4 strongly correlates with muscle invasion, suggesting that patients with high VI-RADS scores may not require repeat TURBT for staging confirmation.^4^,^9^ This can lead to fewer invasive procedures, reduced patient morbidity, and lower healthcare costs while maintaining high diagnostic accuracy.^10^

Pre-operative staging with VI-RADS also facilitates better treatment decision-making. Accurate distinction between MIBC and NMIBC is crucial for determining the appropriate therapeutic approach. Patients with MIBC typically require radical cystectomy or chemoradiotherapy, whereas those with NMIBC can often be managed with intravesical therapy and surveillance.^15^,^16^ By providing a precise, non-invasive assessment, VI-RADS enables clinicians to make informed treatment decisions, potentially improving patient outcomes.^7^,^11^

Furthermore, VI-RADS may serve as an alternative or adjunct to invasive diagnostic methods in specific clinical scenarios. For patients unfit for TURBT or those undergoing surveillance for recurrent tumors, mpMRI with VI-RADS could serve as an alternative for monitoring tumor progression, reducing the need for repeated cystoscopies and biopsies.^6^,^13^

False-negative and false-positive VI-RADS scores carry important clinical consequences. A false-negative interpretation—misclassifying MIBC as NMIBC—could potentially lead to undertreatment and deferred definitive treatment, such as radical cystectomy, increasing the risk of disease progression. Conversely, a false-positive result may lead to overtreatment with radical therapy or chemoradiotherapy in patients without muscle-invasive disease, exposing them to unnecessary morbidity. These scenarios underscore the need to interpret VI-RADS scores in conjunction with histopathology, cystoscopy findings, and patient-specific risk factors. Optimizing cutoff values and standardizing imaging protocols may reduce misclassification and improve patient outcomes.

The use of DCEI is a part of the standard protocol mpMRI protocol in the VI-RADS scoring system; however, its relevance has been increasingly questioned. DCEI improves tumor staging by visualizing early enhancement patterns that help distinguish muscle-invasive from non-muscle-invasive disease. Nevertheless, the administration of gadolinium-based contrast agents poses risks for patients with renal dysfunction and contrast allergies, and DCEI adds complexity to the imaging protocol. Recent reports suggest that bi-parametric MRI (which excludes DCEI and combines only T2WI and DWI) may offer diagnostic performance comparable to contrast-enhanced VI-RADS in detecting MIBC. Incorporating such alternatives may improve the applicability and safety of VI-RADS, particularly in vulnerable patient populations; however, further validation in larger cohorts is needed.

## 5. Limitations of the review

Despite the promising results, this review has several limitations that should be considered. One major challenge is heterogeneity among the included studies, particularly in terms of study populations, MRI protocols, and radiologist experience.^31^,^32^ Some studies exclusively included high-risk NMIBC patients, whereas others focused on newly diagnosed cases, which may have contributed to variability in reported sensitivity and specificity.^33^,^34^ In addition, differences in MRI protocols across institutions remain a concern. While VI-RADS provides standardized scoring guidelines, variations in scanner models and magnetic field strength (1.5T vs. 3T), DWI parameters may affect diagnostic performance.^9^,^18^ The lack of standardized imaging acquisition protocols across institutions could introduce technical bias and impact reproducibility.^19^ Another limitation is the small sample sizes in some studies, which may affect the generalizability of the findings. Studies with fewer participants tend to have wider confidence intervals, leading to greater uncertainty in sensitivity and specificity estimates.^10^,^12^ Future studies should aim to include larger and more diverse patient populations to improve reliability.

## 6. Suggestions for future research

Future research should focus on conducting large-scale, multicenter validation studies to confirm the real-world applicability of VI-RADS. Standardization of mpMRI acquisition protocols across different institutions is crucial to reduce variability in imaging techniques and ensure consistent application of VI-RADS scoring.^3^,^14^,^21^ Further research should explore how radiologist training programs can improve interobserver agreement and enhance the reproducibility of VI-RADS interpretation.^10^ Another promising area of research is the integration of VI-RADS with advanced imaging technologies such as positron emission tomography (PET), computed tomography (CT). In addition, combining VI-RADS with molecular biomarkers could provide a more comprehensive approach to bladder cancer staging and potentially lead to personalized treatment strategies.

## 7. Conclusion

This systematic review highlights the high diagnostic accuracy of VI-RADS in detecting MIBC. Across multiple studies, VI-RADS demonstrated sensitivity values ranging from 78% to 100%, specificity values between 73% and 96%, and AUC values consistently above 0.86. These findings confirm that VI-RADS is a reliable, non-invasive tool for staging bladder cancer. VI-RADS provides a structured and reproducible framework for interpreting mpMRI in the staging of bladder cancer, especially for the evaluation of muscular invasion. Although the diagnostic quality is high in terms of sensitivity, specificity, and interobserver agreement, VI-RADS should not be considered a substitute for pathological confirmation through TURBT or biopsy. Instead, it should be used as an adjunct to facilitate communication between clinicians and radiologists and to support pre-operative planning. Future research should compare VI-RADS not only with clinical examinations such as cystoscopy and TURBT but also with non-standardized MRI interpretations to further validate its added value. The implementation of VI-RADS in clinical practice has the potential to reduce unnecessary procedures and enable earlier detection of MIBC when integrated into existing diagnostic pathways.

## 8. Clinical recommendations

Given its demonstrated diagnostic accuracy, VI-RADS should be integrated as a standardized approach for mpMRI-based staging of bladder cancer in routine clinical practice. Its adoption could minimize unnecessary repeat TURBT procedures, optimize treatment decision-making, and enhance patient outcomes. To ensure consistent real-world application, efforts should focus on radiologist training, MRI protocol standardization, and validation in multicenter settings. Moreover, combining VI-RADS with advanced imaging modalities such as PET-CT and artificial intelligence-driven radiomics may further enhance diagnostic precision. VI-RADS represents a transformative advancement in bladder cancer staging, providing a reproducible, accurate, and non-invasive alternative for distinguishing MIBC from NMIBC. With continued validation and widespread adoption, VI-RADS has the potential to become an essential tool in bladder cancer management, ultimately improving patient care and clinical outcomes.

## Data Availability

Not applicable.
